# Results of First In Vivo Trial of an Acoustic Self-Tonometer

**DOI:** 10.1167/tvst.9.9.18

**Published:** 2020-08-10

**Authors:** Jan Osmers, Oskar Hoppe, Alicja Strzalkowska, Piotr Strzalkowski, Ágnes Patzkó, Stefan Arnold, Michael Sorg, Andreas Fischer

**Affiliations:** 1 University of Bremen, Bremen Institute for Metrology, Automation and Quality Science (BIMAQ), Bremen, Germany; 2 Department of Ophthalmology, University Hospital Würzburg, Würzburg, Germany; 3 Weiss Medizintechnik GmbH, Rednitzhembach, Germany

**Keywords:** self-tonometer, artificial neural network, glaucoma, non-contact

## Abstract

**Purpose:**

Glaucoma is the world's most common cause of irreversible blindness, which makes early diagnosis, with the goal of preserving vision, essential. The current medical intervention is to reduce intraocular pressure (IOP) to slow down progression of the disease. The main goal of this study was to test a novel handheld acoustic self-tonometer on humans.

**Methods:**

A sound pressure pulse generated by a loudspeaker causes the eye to vibrate. A pressure chamber is placed on the human orbit to form a coupled system comprised of the patient's eye, the enclosed air, and the loudspeaker. A displacement sensor in front of the loudspeaker membrane allows the dynamic behavior of the entire system to be detected.

**Results:**

For this clinical trial series, a prototype of the acoustic self-tonometer principle was applied. The resulting membrane oscillation data showed sensitivity of patient IOP, but direct allocation of the measured damping and frequency to the IOP was not significant. For this reason, an artificial neural network was used to find relationships among the subjects’ biometric eye parameters in combination with the self-tonometer data for the IOP reference. An expanded measurement uncertainty (*k_p_* = 2) equal to 6.53 mm Hg was determined for the self-tonometer in a Bland–Altman analysis using Goldmann applanation tonometer reference measurements.

**Conclusions:**

The usability and success rate of producing valid measurement values with the device during self-measurements by test subjects was nearly 92%. The cross-sensitivities observed require compensation in a possible redesign phase to reduce the measurement uncertainty by at least 25% to the maximum of 5 mm Hg required to seek medical device approval.

**Translational Relevance:**

Building on successful laboratory experiments with pig eyes, this article reports the results of testing the acoustic tonometer on humans.

## Introduction

Glaucoma is currently the most common cause of irreversible blindness worldwide.^1^ Because the incidence of glaucoma increases with age, the number of people affected by glaucoma continues to rise also in developed countries. In this disease, neuronal ganglion cells in the optic nerve degenerate due to an undersupply of nutrients, which in advanced stages might lead to blindness.^2^ A pathologically elevated intraocular pressure (IOP) level often occurs in glaucoma patients and is considered one of the major risk factors for glaucoma. Its reduction is a goal of the currently available treatment approaches.^3^ In certain cases, a daily pressure curve may help when choosing the appropriate therapeutic regime.^4^ One goal when determining IOP is to reduce the possible side effects of the measurement process, as measurements are carried out frequently. Further, Goldmann applanation tonometry can only be performed in a doctor's office or at a clinic. Therefore, a non-invasive IOP measurement procedure is desired that can ideally be used by the patient independently in the home environment.

Almost 70 years after its invention, the Goldmann applanation tonometer (GAT) is still the reference tonometer in ophthalmology, due to its low measurement uncertainty (1–2 mm Hg).^5^ However, this measurement has some imperfections; for example, individual biometric data of the cornea can create measurement deviations.^6^^,^^7^ It is necessary to apply eye drops to locally anesthetize the corneal surface, which makes the GAT unsuitable for self-measurements. This is one of the reasons why GAT measurements must be carried out in a doctor's office or clinic. Detailed daily pressure profiles are difficult to obtain, because the number of GAT measurements is limited to in- and outpatient visits.

All tonometers must have a deviation of less than ±5 mm Hg (*k_p_* = 2) compared to GAT readings for medical approval according to ISO 8612:2009. Other contact-based static tonometers are the Schiötz tonometer and the Mackay–Marg tonometer.^8^^,^^9^ These methods have disadvantages similar to those for the GAT and show a higher measurement uncertainty than the GAT.^8^^,^^9^

The iCare rebound tonometer (iCare Finland Oy, Vantaa, Finland), which shoots a small plastic-covered metal pin onto the cornea from a short distance and measures the rebound velocity, is also contact based but does not require an anesthetic. The measurement uncertainty is higher in comparison to the GAT, with a confidence interval of ±6.6 mm Hg (*k_p_* = 2) within the physiological normal pressure range.^10^ Above 23 mm Hg, the measurement uncertainty is even greater.^11^ It is difficult to achieve precise IOP readings with the iCare HOME version, and it has a higher measurement uncertainty than the iCare Pro in tests.^12^

In addition to contact tonometers, non-contact air pulse tonometers are frequently used for ambulatory IOP measurement. This measurement method uses a short air pulse to apply pressure to the cornea from a short distance. The time that elapses until the cornea flattens out is taken as a measure of IOP. The measurement uncertainty of ±4 mm Hg for air pulse tonometers is higher than for the GAT.^13^ In the case of corneal changes, far higher measurement deviations are possible. For this reason, air pulse tonometers are mainly used for screening. The deformation of >1 mm^14^ of the eye is enormous compared to GAT measurements, and the principle does not seem suitable for self-measurement. Anxious defensive reactions of the patient cause additional measurement deviations, and there is also a risk of infection due to spraying tear fluid.

For the GAT, measurements at the required frequency (>5 per day^4^^,^^15^^,^^16^) are associated with considerable effort and must be performed, in most cases, under inpatient conditions. These measurements cannot be performed by the patients themselves. For this reason, there is a clear demand for a contactless, gentle device for measuring IOP that can be used by the patients themselves in the home environment.

Another tonometric approach analyzes the vibration characteristics of the eye, because the frequency of the eye's eigenmodes after acoustic excitation depends on the IOP.^17^^–^^19^ The challenge with these approaches is to identify and correct the cross-sensitivities with respect to the biometric parameters, eye movement, and pulsation of the eye.^18^ Although the results looked promising during the research phase, none of the approaches made it to the market and no ophthalmological device utilizing this principle is available to date.

Another approach to acoustic tonometry was presented by Freyberg et al.,^20^ who placed a pressure chamber on the orbit and used a loudspeaker to excite the eye to vibrate. A statistical relation between the pressure measured inside the chamber and the IOP was determined. Osmers et al.^21^ tested this acoustic tonometry approach on porcine eyes and presented a physical model explaining the measurement principle. It was found that the damping of a coupled system consisting of a loudspeaker, a closed pressure chamber, and a connected eye can produce a sensitive IOP value. The damping was determined directly on the membrane of the loudspeaker with a light reflection sensor. The promising results of the laboratory experiments with a measurement uncertainty of <2 mm Hg prompted further investigation into the achievable measurement uncertainty of this method when applied to humans.^21^

For this reason this measurement approach has been tested on humans to determine the achievable measurement uncertainty in humans and to prove the applicability of the device. The methods section presents the prototype of the acoustic self-tonometer and how the data was acquired. The results section provide the results of the clinical trial including statistics regarding the eye parameters statistics regarding the eye parameters of the participants, an overview of the usability of the acquired data, and results of the artificial neural network. A brief discussion of identified cross-sensitivities and limitations is also provided.

## Materials and Methods

### Acoustic Self-Tonometer

**Figure 1.**
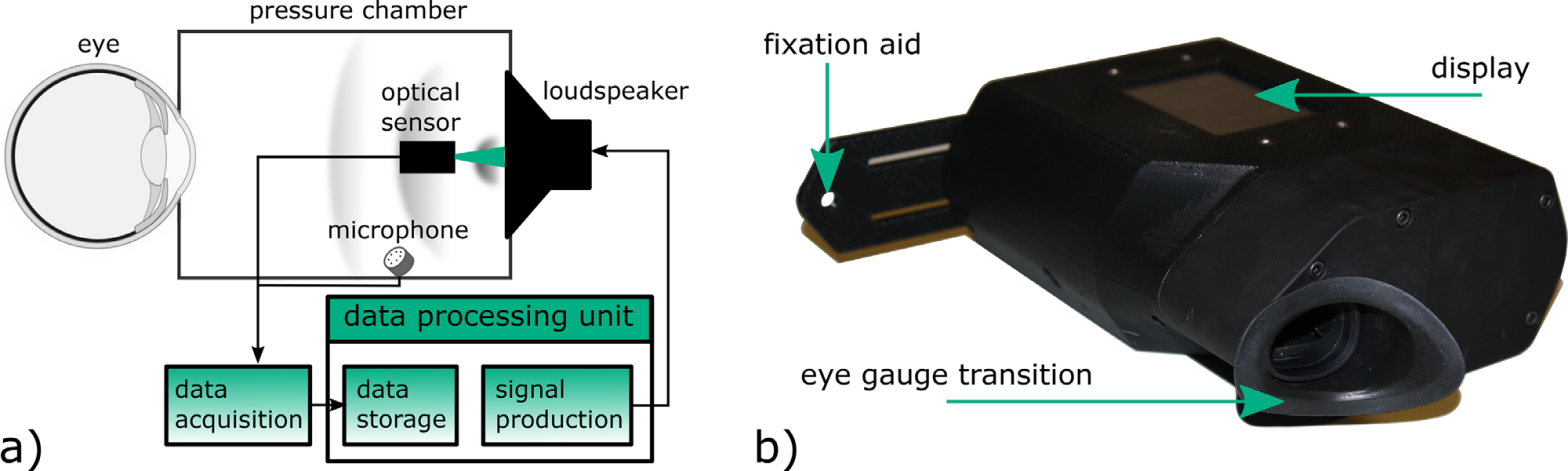
(a) Schematic working principle of acoustic self-tonometer. (b) Prototype of self-tonometer that was used for data acquisition on humans in the clinical trial series.

In order to test the acoustic tonometry approach on humans, a handheld self-tonometer was created that was similar to the laboratory test setup described in Osmers et al.^21^ The measurement approach requires a closed pressure chamber that fits onto the orbit of the patient to create a coupled mechanical system consisting of the eye to be measured, the gas volume, and the loudspeaker. For this reason, a silicon eye gauge transition was designed to seal the orbit and fit on the right and the left eye. Figure 1 shows the schematic working principle and the prototype handheld self-tonometer that was used for the clinical trials to acquire measurement data and to test the applicability. According to the acoustical measurement principle, a sound pressure excitation is generated by a 30-mm-diameter loudspeaker that amounts to approximately 250 Pa. The vibration of the loudspeaker membrane is detected by a light reflection sensor. The output signal of the reflection sensor is processed further to quantify the change of the oscillation behavior of the coupled mechanical system, which, after calibration, is used to calculate the IOP value of the patient.

### Study Design

The study was approved by the local ethics committee. The 96 test participants (49 female, 47 male; age, 62 ± 17 years) were recruited on a ward as well as in the outpatient clinic of the Department of Ophthalmology at the University of Würzburg between September 2016 and December 2017. They all signed an informed consent. To exclude possible factors that could potentially lead to variability in this early stage of the development of a new tonometry device, stringent exclusion criteria were applied: patients <18 years, patients with astigmatism >2 diopters, or patients with pathological corneal deformations, such as keratoconus or keratoglobus, as determined with the Pentacam system (OCULUS Optikgeräte GmbH, Wetzlar, Germany), for whom GAT measurement values could not be reliably acquired. Further exclusion criteria included any surgical intervention within 3 months of consent to the clinical trial or acute inflammation or injury to the ocular surface. The clinical trials were conducted in accordance with Good Clinical Practice guidelines and adhered to the tenets of the Declaration of Helsinki. The test participants’ data were pseudonymized by the hospital staff during the survey, and the pseudonymized data were made available by the clinic for evaluation. Note that the experiments involved only the acquisition of measurement data on patient with associated GAT reference measurements for development of the signal evaluation algorithms. This was not an approval study of a fully developed tonometer.

The IOP of all patients was measured with GAT. Therapeutic decisions were made on the basis of the GAT measurement, independent of the self-tonometer measurement. Outpatients performed a single measurement with the self-tonometer, and inpatients performed up to four measurements during the day and one measurement at night while lying down as part of a daily pressure curve measurement. The measurement with the self-tonometer was always carried out before the reference measurement, as the cornea is flattened by GAT measurement and deformed with a deformation distance of about 152 µm for >1 second, which can subsequently affect IOP for several minutes.^22^ An influence of the self-tonometer on the IOP can be neglected due to the lower deformation of the eye with a deformation distance of approximately <80 µm for less than 10 ms.^23^ In addition, contactless recordings of biometric eye parameters were made before the first measurement. The central corneal thickness and curvature was measured with the Pentacam system, and axis length was measured with the IOLMaster (Carl Zeiss Meditec AG, Jena, Germany).

After brief instruction on how to use the self-tonometer, the patients carried out self-measurements. At this developmental stage, only the raw data were stored on a USB stick for further evaluation, and no IOP results were displayed on the display of the device. A light on the self-tonometer confirmed successful recording of the measurement data, so patients were able to perform another measurement in case of an unsuccessful recording.

### Data Acquisition and Evaluation

The saved data were transferred from the USB stick in the self-tonometer to a PC. The evaluation software was programmed with the software MATLAB (MathWorks, Natick, MA). Due to test subject movement during measurement and the resulting pressure fluctuations inside the chamber, data preprocessing was required. The varying offset of the measurement signal was balanced by an interpolation using third-order polynomials, which provided stable signal levels and allowed for an averaging of the approximately 20 individual signal responses of the repeated excitation pulses within one measurement. The characteristic values of these averaged signal responses, particularly damping and frequency, were calculated with the corresponding standard deviation. Additionally, artificial neural networks (ANNs) were used for the data evaluation to combine biometric eye parameters and the measured curve parameters of the self-tonometer. ANNs have the ability to assign a large number of input parameters to an output variable and to recognize a mathematical relationship.

A decisive influencing factor for the results of the ANN is the architecture of the network (i.e., the number and networking of the internal switching units, the so-called neurons).^24^ In principle, ANNs have one layer of input neurons, whereby one neuron exists for each intended input parameter. This is followed by any number of neurons divided into any number of hidden levels.^24^ Finally, there is an output neuron that summarizes and outputs the result value. The mathematical relationship between the input parameters and the output parameter is learned in the training phase of the ANN. Here, a share of the data pool is separated to allow for validation with unknown data after a mathematical relationship is found. It should be noted that the number of inner neurons also determines the degrees of freedom that the net has available for calculating the result, similar to the order of a polynomial. With too many degrees of freedom, so-called “overfitting” can occur, whereby the ANN maps the training data of the iterative process very well, but at the same time loses its generalization ability. It is therefore not possible to calculate unknown input data with new combinations of values, even if they represent comparable correlations. This results in an inaccurate mapping of the separated validation data. For this reason, great effort was put into investigating the best architecture of the ANN for the allocation of the self-measurements and the biometric data to the GAT reference values.

## Results

The aim of the study was to test an acoustic measurement approach on humans with known IOP reference values determined with a GAT, which is the current gold standard for IOP measurements. The hypothesis postulated in the study is that the response of the human eye to a vibration excitation varies as a function of IOP and that this can be detected using the measurement principle presented in Osmers et al.^21^ Because the vibration response can also be influenced by environmental conditions (physiological conditions), it was crucial to identify and monitor relevant influencing factors in this first trial.

### IOP and Eye Parameters of the Participants

An overview of the measured biometric data is provided in Table 1. Except for age and IOP values, the mean dimensions are representative for average human eyes.^14^^,^^25^^,^^26^ Both age and IOP are elevated, as is common among glaucoma patients.^1^

**Table 1.** Eye Parameters of the 96 Test Subjects (*k_p_* = 1)

	Mean ± SD	
Parameter	Right Eye (OD)	Left Eye (OS)
Age, y	61.7 ± 17.1	—
IOP (GAT), mm Hg	16.4 ± 4.4	17 ± 5.7
Visual acuity	0.7 ± 0.3	0.7 ± 0.3
Cornea radius, mm	7.8 ± 0,3	7.8 ± 0.3
Central corneal thickness, µm	546.9 ± 36.4	544 ± 37,6
Axial length, mm	24 ± 3.1	24.2 ± 1.7
Anterior chamber		
Anterior chamber depth, mm	3.1 ± 0.7	3.1 ± 0.7

### Usability of Raw Data

**Figure 2.**
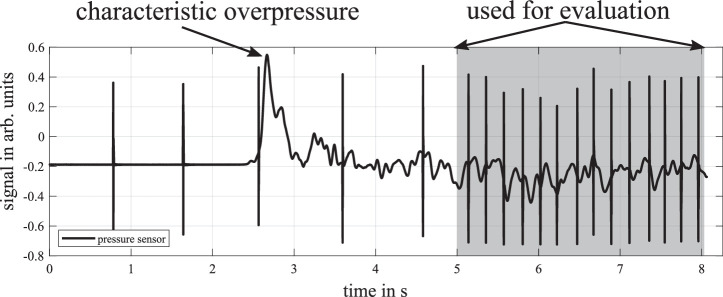
Course of raw pressure signal with characteristic touchdown behavior leading to overpressure at 2.8 seconds. The pre-evaluation phase from 0 to 5 seconds is not further evaluated. Starting from 5 seconds, the impulses were evaluated for IOP estimation (*gray area*). The pressure chamber was considered to be airtight.

The automated evaluation requires an initial evaluation of the validity of the dataset. A pressure fluctuation is expected when the self-tonometer is placed correctly on the orbit, as the volume is slightly compressed by the flexible eye gauge transition and the pressure rises according to the general gas equation (pressure sensor signal in Fig. 2). The measurements with and without this touchdown pattern are compared with respect to the curve parameters of damping and frequency of the measurement pulses. In our study, after visual categorization of the two touchdown patterns, the damping and frequency curve parameters were significantly different in both measurement situations. It is assumed that the measurement with the touchdown pattern before the start of the measurement was successful. But, movement by a test subject during the ongoing measurement can result in unusable data; for example, single signal pulses can exceed the range of the sensor due to movement-related pressure changes within the chamber. In order to determine a successful measurement without visual inspection, expectation ranges were defined for the curve parameters. The individual signal pulses must have at least seven zero crossings, the pulses must have a frequency from 350 to 570 Hz, and at least three valid pulses must be measured.

Table 2 provides an overview of the usability of the measurement data generated during the clinical study of the acoustic self-tonometer instrument. Based on the predetermined requirements, 504 of the 828 datasets could be used for further evaluation. Of the 324 measurements excluded from the study, 56 did not start with a short pulse sequence, four showed unusually large pressure fluctuations, and 264 did not provide enough valid measurement pulses for statistical evaluation. Using the prototype of the self-tonometer, the subjects were able to carry out measurements on their own in 91.6% of the documented self-measurements, of which 68.3% could be used for further evaluation given the established quality standards.

**Table 2.** Success Rate of Measurements

Measure	Number	Share (%)
Total number of records	828	100
Expectancy-compliant data	504	60.9
No start of measurement	56	6.8
Quality standard not met	264	31.9
Pressure deviation	4	0.5

Test subjects performed 626 measurements themselves; 202 measurements were supported by a medic.

Laboratory tests with porcine eyes have shown that IOP can be significantly correlated to damping of the system, at least for each single eye.^21^ The standard deviation of the damping of this system for the repeated single impulses in each of the self-tonometer measurements amounted to 2.1% on average, indicating good reproducibility despite the test subject's movements while holding the device onto the orbit.

The measurements of the clinical trials were compared both intra- and inter-individually. The patient-wise comparison only partially showed clearly attributable differences. Frequently, a distinction was found between signal damping and frequency, even during measurements with the same reference IOP, which is undesirable. However, the number of measurements for each individual and the span between measured IOP values were generally too small to identify significant trends. Even in the overall comparison of all measurements, the expected sensitivity in the characteristic damping and frequency values of the recorded pulse cannot be clearly correlated to the reference IOP.

For this reason, we investigated potential causes for the differences between the laboratory tests and the clinical study. As a first approach for further data evaluation, the acquired biometric data had to be included to compensate for unique differences in the test subjects’ eye geometry. This approach also created a broader bandwidth of IOP values, as the measurements of all subjects were taken into account for one entire data pool to be used for the development of the evaluation algorithm. In order to include the individual biometric parameters of the test subjects in the evaluation, parameter changes in nine dimensions had to be considered to confirm the assumed dependence of the damping or frequency of the system on IOP. This challenge was met by the use of an ANN.

### Artificial Neural Network Evaluation Results

ANN architectures with two hidden layers and five to 13 neurons in each of the hidden layers were calculated. The evaluation of the ANN result was performed according to the inclination of the regression line and the regression coefficient of the correlation from GAT results and ANN output. Both parameters improved with a rising number of neurons. The choice of the final architecture was made considering the determined measurement uncertainty for test data that were not used in the iterative training process and is therefore comparable to new data and allows for validation of the ANN.

The resulting ANN has nine input parameters, two hidden layers, and one output neuron that gives IOP values. The following results for a net architecture with seven neurons in the first hidden layer and 10 neurons in the second hidden layer of the ANN show a statistically low measurement uncertainty for the test data.

**Figure 3.**
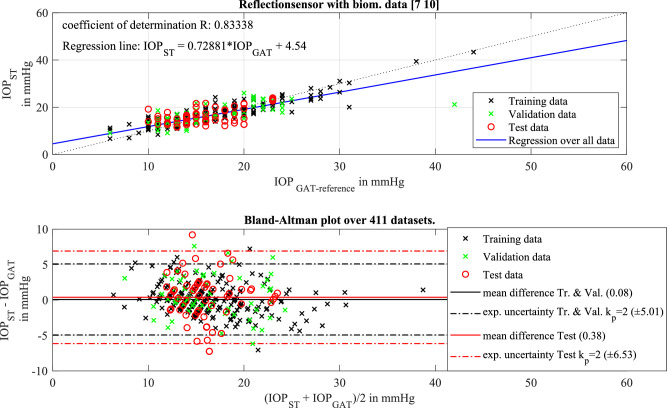
(*Top*) Regression plot of the ANN result. (*Bottom*) Bland–Altman plot of the self-tonometer results (ST) in reference to the GAT measurements. The achieved measurement uncertainty is calculated only from test data that was not used in the iterative ANN training and amounts to 6.53 mm Hg (*k_p_* = 2).

The results from the ANN evaluation are presented in two ways. First, the correlation between the GAT reference and the output of the ANN as a self-tonometer result is used, where the inclination of the regression line is a value for the sensitivity of the ANN results. The regression itself shows the scattering of the values. The ANN evaluation was repeated in order to overcome the limitations of local minima during the iterative training process. The upper graph in Figure 3 shows the correlation between the self-tonometer results from the ANN and the GAT reference values. The regression value amounts to 0.83, which is in good agreement with the data for the regression line that indicates the sensitivity of the correlation. The inclination is nearly 0.73. For a medical interpretation, the results of the two tonometric measurements were compared in a Bland–Altman plot. Here, the expanded measurement uncertainty of the self-tonometer amounts to 6.53 mm Hg (*k_p_* = 2) with respect to the GAT reference. This measurement uncertainty was only calculated for the test values of the ANN used for testing the already trained ANN. They have no influence on the training and are therefore regarded as “new” measurements.

Because the ANN is an iterative evaluation process that suffers from local minima or maxima of the determined mathematical relation, a statistical analysis was conducted over the results of 100,000 different training trials with the same ANN architecture. The results shown in Figure 3 are representative of the average of the 1000 ANNs that best achieved the combination of slope of regression line and regression coefficient. The average slope of the regression lines is 0.65 ± 0.04 mm Hg/mm Hg, and the average expanded uncertainty (*k_p_* = 2) from the Bland–Altman plots for the entire dataset is 6.0 mm Hg. Thus, there is a significant correlation of the measurement data recorded by the self-tonometer in combination with the biometric measurements to the IOP values measured with the GAT reference.

## Discussion

The achieved measurement uncertainty of 6.53 mm Hg must be reduced in order to receive medical device approval. Nevertheless, it is a promising first in vivo trial of the acoustic self-tonometer that provides data about the usability of the device by elderly people, as well as valuable information for the redesign phase. To reduce the measurement uncertainty, the identified cross-sensitivities of the tonometer principle and setup must be adjusted. The tightness of the pressure chamber when connected to the human orbit is a crucial factor affecting the reliability of measurement results; however, no sensor concept was equipped that checked for leakage of the pressure chamber at the required order of magnitude in the presented first trial.

In a subsequently performed test by two technically educated test subjects who paid particular attention to maintaining a tight pressure chamber for their 32 measurements, measurement uncertainty was reduced to 2.0 mm Hg for the ANN developed from the clinical trial. Particularly for the self-measurements of the patients, the data corrupted by leakage of the pressure chamber cannot be identified; consequently, in the redesign phase of the self-tonometer, a sensor must be integrated to reliably detect any leakage.

For future evaluations with an improved device that is able to compensate for the identified cross-sensitivities, it will be important to mask investigators recording IOPs with the GAT to reduce the risk of bias in the ANN. Furthermore, a larger proportion of measurements with low IOP values (<11 mm Hg) and high IOP values (>25 mm Hg) is desirable for the next series of clinical trials in order to better define measurement uncertainty in the boundary regions.

## Conclusions

To our knowledge, this was the first acoustic measurement approach tested on humans in a clinical trial series. The applicability of the tonometer as self-tonometer for home use was confirmed, as 626 measurements were performed by the patients themselves with a success rate of nearly 92%. The identified cross-sensitivities, such as leakage of the pressure chamber and patient-specific biometric influences, must be considered in future research in order to lower the determined measurement uncertainty by more than 25% to fall below the 5 mm Hg required for medical device approval. Such a self-tonometer may be of great benefit to glaucoma patients, due to its potential for patients to take their own non-invasive home measurements, from which daily curves of the IOP can easily be created. The recorded daily curves of the IOP could provide improved assessment of the success of therapeutic IOP reductions and therefore support medical therapy.

## References

[1] Jonas JB, Aung T, Bourne RR, Bron AM, Ritch R, Panda-Jonas S Glaucoma. *Lancet*. 2017; 390: 2183–2193. 28577860 10.1016/S0140-6736(17)31469-1

[2] Grehn F *Augenheilkunde*. Berlin, Germany: Springer; 2008.

[3] Fry LE, Fahy E, Chrysostomou V, et al. The coma in glaucoma: retinal ganglion cell dysfunction and recovery. *Prog Retin Eye Res*. 2018; 65: 77–92. 29631042 10.1016/j.preteyeres.2018.04.001

[4] Arora T, Bali SJ, Arora V, Wadhwani M, Panda A, Dada T Diurnal versus office-hour intraocular pressure fluctuation in primary adult onset glaucoma. *J Optom*. 2015; 8: 239–243. 26386536 10.1016/j.optom.2014.05.005 PMC4591418

[5] Goldmann H, Schmidt TH Applanation tonometry. *Ophthalmologica*. 1957; 134: 221–242. 13484216 10.1159/000303213

[6] Tonnu PA, Ho T, Newson T, et al. The influence of central corneal thickness and age on intraocular pressure measured by pneumotonometry, non-contact tonometry, the Tono-Pen XL, and Goldmann applanation tonometry. *Br J Ophthalmol*. 2005; 89: 851–854. 15965165 10.1136/bjo.2004.056622 PMC1772720

[7] Khan MA Numerical study on human cornea and modified multiparametric correction equation for Goldmann applanation tonometer. *J Mech Behav Biomed Mater*. 2014; 30: 91–102. 24269944 10.1016/j.jmbbm.2013.10.002

[8] Mackay RS, Marg E Fast, automatic, electronic tonometers based on an exact theory. *Acta Ophthalmol (Copenh)*. 1959; 37: 495–507. 14419489 10.1111/j.1755-3768.1959.tb03461.x

[9] Rosa N, Lanza M, Cennamo G, et al. Accuracy of Schiotz tonometry in measuring the intraocular pressure after corneal refractive surgery. *J Optom*. 2008; 1: 59–64.

[10] Grigorian F, Grigorian AP, Li A, Sattar A, Krishna R, Olitsky SE Comparison of the Icare rebound tonometry with the Goldmann applanation tonometry in a pediatric population. *J AAPOS*. 2015; 19: 572–574. 26691047 10.1016/j.jaapos.2015.08.009

[11] Munkwitz S, Elkarmouty A, Hoffmann EM, Pfeiffer N, Thieme H Comparison of the iCare rebound tonometer and the Goldmann applanation tonometer over a wide IOP range. *Graefes Arch Clin Exp Ophthalmol*. 2008; 246: 875–879. 18196259 10.1007/s00417-007-0758-3

[12] Mudie LI, LaBarre S, Varadaraj V, et al. The Icare HOME (TA022) study: performance of an intraocular pressure measuring device for self-tonometry by glaucoma patients. *Ophthalmology*. 2016; 123: 1675–1684. 27289178 10.1016/j.ophtha.2016.04.044

[13] Cook JA, Botello AP, Elders A, et al. Systematic review of the agreement of tonometers with Goldmann applanation tonometry. *Ophthalmology*. 2012; 119: 1552–1557. 22578443 10.1016/j.ophtha.2012.02.030

[14] Huseynova T, Waring GO, Roberts C, Krueger RR, Tomita M Corneal biomechanics as a function of intraocular pressure and pachymetry by dynamic infrared signal and Scheimpflug imaging analysis in normal eyes. *Am J Ophthalmol*. 2014; 157: 885–893. 24388837 10.1016/j.ajo.2013.12.024

[15] Drance SM Diurnal variation of intraocular pressure in treated glaucoma. Significance in patients with chronic simple glaucoma. *Arch Ophthalmol*. 1963; 70: 302–311. 14048787 10.1001/archopht.1963.00960050304004

[16] Asrani S, Zeimer R, Wilensky J, Gieser D, Vitale S, Lindenmuth K Large diurnal fluctuations in intraocular pressure are an independent risk factor in patients with glaucoma. *J Glaucoma*. 2000; 9: 134–142. 10782622 10.1097/00061198-200004000-00002

[17] Drescher J *Bestimmung des Intraoculardrucks aus dem Schwingungsverhalten des Humanauges*. Karlsruhe, Germany: Karlsruhe Institute of Technology 2000. Thesis.

[18] Gundlach A *Interferometrische Schwingungsanalyse des menschlichen Auges zur Bestimmung des Intraokulardrucks*. Karlsruhe, Germany: Karlsruhe Institute of Technology 2003. Thesis.

[19] Hey S *Berührungslose Anregung und Analyse von Schwingungen des menschlichen Auges zur Frühdiagnose einer Glaukomerkrankung*. Karlsruhe, Germany: Karlsruhe Institute of Technology 2003. Thesis.

[20] A von Freyberg, M Sorg, Fuhrmann M, et al. Acoustic tonometry: feasibility study of a new principle of intraocular pressure measurement. *J Glaucoma*. 2009; 18: 316–320. 19365198 10.1097/IJG.0b013e3181845661

[21] Osmers J, Á Patzkó, Hoppe O, Sorg M, Freyberg A, Fischer A The influence of intraocular pressure on the damping of a coupled speaker-air-eye system. *J Sens Sens Syst*. 2018; 7: 123–130.

[22] Wilke K Effects of repeated tonometry: genuine and sham measurements. *Acta Ophthalmol*. 1972; 50: 574–582. 4678278 10.1111/j.1755-3768.1972.tb05987.x

[23] Osmers J, Sorg M, Fischer A Optical measurement of the corneal oscillation for the determination of the intraocular pressure. *Biomed Tech (Berl)*. 2018; 64: 471–480. 10.1515/bmt-2018-0093 30231007

[24] Kroll A *Computational Intelligence: Eine Einführung in Probleme, Methoden und Technische Anwendungen*. Munich, Germany: Oldenbourg; 2013.

[25] Brandes R, Lang F, Schmidt RF *Physiologie des Menschen*. Berlin, Germany: Springer; 2019.

[26] Sobotta J, Putz R, Pabst R *Anatomie des Menschen der komplette Atlas in einem Band; allgemeine Anatomie, Bewegungsapparat, innere Organe, Neuroanatomie*. Munich, Germany: Elsevier; 2007.

